# Serum Lipid Profile of Newly Diagnosed Hypertensive Patients in Nnewi, South-East Nigeria

**DOI:** 10.1155/2012/710486

**Published:** 2012-12-04

**Authors:** Charles U. Osuji, Emeka G. Omejua, Emmanuel I. Onwubuya, Gladys I. Ahaneku

**Affiliations:** ^1^Department of Medicine, Nnamdi Azikiwe University Teaching Hospital, PMB 5025, Nnewi 435101, Anambra State, Nigeria; ^2^Department of Medicine, Federal Medical Centre, P.O. Box 1010, Owerri 460281, Imo State, Nigeria

## Abstract

Abnormalities in serum lipid and lipoprotein levels are recognized major modifiable cardiovascular disease and essential hypertension risk factors. The objective of this study was to examine the serum lipid patterns of newly diagnosed hypertensive patients attending a tertiary healthcare centre in South East Nigeria. *Methods*. Two hundred and fifty newly diagnosed adult hypertensive patients and an equal number of age- and sex-matched controls without hypertension were consecutively recruited from the Medical and General Outpatient Clinics of Nnamdi Azikiwe University Teaching Hospital, Nnewi. *Result*. 126 males and 124 females were in each of the two groups. Mean age was comparable in both groups. Hypertensives had significantly higher mean systolic blood pressure, diastolic blood pressure, body mass index, waist circumference, waist-hip ratio, and fasting blood sugar than the controls. The mean TC, TG, and LDL-C were significantly higher among the hypertensives. The mean HDL-C was comparable; *P* = 0.8. Among the hypertensive subjects, there was statistically significant positive correlation between BMI and TC; LDL-C and TG; WC and TG; FBS and TC; LDL-C and TG. HDL-C showed a statistically significant inverse correlation with WHR in hypertensives. *Conclusion*. This study showed that lipid abnormalities are highly prevalent among newly diagnosed hypertensives in South-East Nigeria.

## 1. Introduction

Abnormalities in serum lipid and lipoprotein levels (dyslipidemia) are recognized as major modifiable cardiovascular disease (CVD) risk factors [1] and have been identified as independent risk factors for essential hypertension giving rise to the term dyslipidemic hypertension [2, 3].

Dyslipidemia is more common in untreated hypertensives than normotensives, and lipid levels increase as BP increases [4, 5]. Though no specific pattern of dyslipidemia has been consistently reported among hypertensive individuals, many studies have shown that total cholesterol (TC), triglycerides (TG), and virtually all fractions of lipoproteins tend to be more frequently abnormal among hypertensive patients than in the general population. In general, black Africans have been reported to have lower serum total cholesterol and higher high-density lipoprotein cholesterol (HDL-C) than whites and other blacks in industrialized countries; however, as in Westernized countries, age, sex, socioeconomic status, and diet also significantly affect lipid levels in healthy Africans [6–9].

In Nigeria although the incidence of coronary artery disease and atherosclerosis is still low, it is rising as atherosclerotic lesions of the aorta, coronary, and cerebral arteries are being reported [10, 11]. Hypertension is a powerful risk factor for cardiovascular disease and it remains one of the biggest health and economic issues facing the world [12, 13] and in Nigeria the prevalence of hypertension is known to have varied from 11% to 45% [14–16].

Hypertension is known to be associated with alterations in lipid metabolism which gives rise to abnormalities in serum lipid and lipoprotein levels. It has also been documented that presence of hyperlipidaemia substantially worsens the prognosis in hypertensive patients [17]. 

The frequent clustering of hypertension, lipid abnormalities, and other metabolic abnormalities, in an individual has been clearly demonstrated to be synergistic in accelerating atherosclerosis and development of CVD [18]. With the current trend of increasing incidence and prevalence of hypertension, CVD, and other noncommunicable diseases coupled with the persistence of high rates of communicable diseases in most developing countries, these countries have been said to be experiencing a “double burden of disease.”

The objective of this study was to examine the serum lipid patterns of newly diagnosed hypertensive patients attending a tertiary healthcare centre in South East Nigeria.

## 2. Materials and Methods

Two hundred and fifty newly diagnosed adult hypertensive patients and an equal number of age- and sex-matched controls without hypertension were consecutively recruited from the Medical and General Out-patient Clinics of Nnamdi Azikiwe University Teaching Hospital, Nnewi, were studied. It was a hospital-based cross-sectional study. Relevant sociodemographic data and history were obtained, physical examination was carried out, and anthropometric measurements were taken during subjects' first visits to the clinics. Blood pressure was taken on the left arm after 5 minutes' relaxation, in a sitting position, using a standard mercury sphygmomanometer with appropriate cuff size; systolic (SBP) and diastolic (DBP) blood pressures corresponded to Korotkoff sounds 1 and V, respectively. The average of three readings, taken at first visit, was used for further analysis. Height and body weight were measured with participants standing without shoes and heavy outer garments. Body mass index (BMI) was calculated as weight, divided by height squared (kg/m^2^). Hip and waist were measured to the nearest 1 cm and waist-to-hip ratio (WHR) was calculated as waist circumference divided by hip circumference. Fasting blood glucose and fasting serum lipid profile were determined using a 12-mL sample of blood obtained from an antecubital vein following an overnight (i.e., about 9–12-hour) fast. Serum total cholesterol (TC), high density lipoprotein cholesterol (HDL-C), and triglycerides (TG) were determined enzymatically, while low density lipoprotein cholesterol (LDL-C) was calculated using the Friedwald formula. 

Hypertension was diagnosed based on the JNC-7 criteria [19]. An informed signed consent was needed to be recruited into the study and those who refused were not recruited into the study. Hypertensive subjects who were already on antihypertensive medications and those with known or suspected secondary hypertension were not recruited. Other exclusion criteria were: prior diagnosis of diabetes mellitus, pregnancy, current or recent intake of drugs such as statins, beta blockers, and so forth that affect lipid metabolism. Serum total cholesterol (TC), low-density lipoprotein cholesterol (LDL-C), high density lipoprotein cholesterol (HDL-C), and triglyceride (TG) levels were classified on the basis of the Third Report of the Expert Panel on Detection, Evaluation, and Treatment of High Blood Cholesterol in Adults (ATP III) [18]. Elevated TC was defined as having TC levels of  >5.17 mmol/L (200 mg/dL). Low HDL-C was defined as having HDL-C levels <1.03 mmol/L (< 40 mg/dL) elevated LDLC was defined as having LDL-C levels of >3.38 mmol/L  (>130 mg/dL). Elevated TG was defined as having triglyceride levels of >1.69 mmol/L  (>150 mg/dL).

Ethical clearance was obtained from the Nnamdi Azikiwe University Teaching Hospital Ethical Committee (NAUTHEC).

## 3. Statistical Analysis

Data was analyzed using the Statistical Package for Social Sciences (SPSS) version 16.0 software (SPSS Inc., Chicago, IL, USA). Simple descriptive statistics was used to present the demographic characteristics of the study participants. Continuous variables were presented as mean ± standard deviation and were compared using the student *t*-tests while Chi-square (*χ* ^2^) tests were used to compare categorical variables. Other associations were evaluated with Spearman's correlation coefficient as well as multiple linear regression analysis. A *P* value of <0.05 was considered statistically significant.

## 4. Results

The clinical and biochemical characteristics of the study participants are shown in Table 1. The hypertensive subjects and the controls were matched for age and sex. Among each of the 2 groups, there were 126 males and 124 females (1.02 : 1). The mean age of the subjects (58.5 ± 12.4 years) was comparable to that of the controls (57.8 ± 12.5 years). The hypertensive subjects had significantly higher mean systolic blood pressure (SBP), diastolic blood pressure (DBP), body mass index (BMI), waist circumference (WC), waist-hip ratio (WHR), and fasting blood sugar (FBS) than the controls. The mean TC (4.83 ± 0.95 versus 4.15 ± 0.57, *t* = −9.70, *P* < 0.001), TG (1.23 ± 0.32 versus 1.10 ± 0.24, *t* = −5.37, *P* < 0.001), and LDL-C (3.00 ± 0.82 versus 2.44 ± 0.53, *t* = −9.11, *P* < 0.001) were also significantly higher among the hypertensive subjects. The mean HDL-C was however comparable in the two groups (1.25 ± 0.27 versus 1.24 ± 0.04, *t* = −0.25, *P* = 0.8).

Table 2 shows the prevalence of various lipid abnormalities (based on the NCEP ATP III criteria). Among the hypertensive subjects; 131 (52.4%) had at least one abnormal lipid parameter while 56 (22.4%) of the controls had at least one lipid abnormality. Elevated TC was the most frequently occurring abnormality among the hypertensive subjects, *n* = 89 (35.6%), followed by elevated LDL-C, *n* = 71 (28.4%). These abnormalities however often occurred together with other lipid abnormalities rather than in isolation. Participants with isolated low HDL-C (hypertensives, *n* = 54 (21.6%); controls, *n* = 40 (16.0%)) represented 41.2% and 71.6% of all hypertensives subjects and controls with dyslipidemia respectively. Isolated low HDL-C was therefore the most frequent individual lipid abnormality among both the hypertensive subjects and controls.  Table 3 shows the Spearman's correlation coefficient between the serum lipid indices and various clinical and biochemical parameters.  Among the hypertensive subjects, there was a statistically significant positive correlation between BMI and TC (*r* = 0.176, *P* = 0.005); BMI and LDL-C (*r* = 0.176, *P* = 0.005); BMI and TG (*r* = 0.288, *P* < 0.001); WC and TG (*r* = 0.293, *P* < 0.001); FBS and TC (*r* = 0.168, *P* = 0.008); FBS and LDL-C (*r* = 0.172, *P* = 0.006); FBS and TG (*r* = 0.136, *P* = 0.03). On the other hand, HDL-C showed a statistically significant inverse correlation with WHR (*r* = −0.241, *P* < 0.001) among the hypertensive subjects. Among the control, there was no statistically significant correlation between the lipid indices and any of the above clinical (SBP, DBP, BMI, WC, WHR) or biochemical (FBS) parameters.  Table 4 shows the multiple regression analysis with total cholesterol, HDL-C, LDL-C, and triglycerides as dependent variables and BP, age, BMI, sex, and FBG as independent variables.

## 5. Discussion

In this study, serum TC, TG, and LDL-L concentrations are significantly higher in hypertensive patients than in normotensive subjects. This is consistent with earlier observations in parts of the world and in other parts of Nigeria [17, 20–25]. This is unlike the findings of Akintunde [26], Lepira et al. [27] and Kesteloot et al. [28] who reported that the TC, TG, and LDL-C of newly diagnosed hypertensive patients did not differ significantly from that of control subjects, though the newly diagnosed hypertensive tended to have a higher level of LDL-C, TG, TC.

In our study, serum TC concentrations are significantly higher in hypertensive patients than in normotensive subjects. This is consistent with earlier observations in parts of the world and in other parts of Nigeria [17, 20–24, 29]. High levels of serum cholesterol are known to increase the risk of developing macrovascular complications such as coronary heart disease (CHD) and stroke [30]. Many epidemiological studies indicate a progressive increase in CHD risk as the serum TC exceeds 5.0 mmol/L [31] which prompted Lewis [32] to suggest that levels of serum TC in the range 5.0–6.5 mmol/L to be considered undesirable. It is to be noted that there was positive and significant correlation between serum TC and both systolic and diastolic BP in both hypertensive patients and normotensive controls. Similarly, there were statistically significant correlations between serum TC and BMI among both hypertensive and normotensive groups. The hypertensive patients had significantly higher BMI and WHR than the controls. This observation may be due to common risk factors for hypertension, obesity and dyslipidaemia as obesity, is known to play a central role in the causation and sustenance of insulin resistance [20], though our study was a cross-sectional study. The exact pathogenetic mechanisms underlying the CVD risk mediated by dyslipidemia are not fully elucidated, but high levels of serum cholesterol are known to increase the risk of developing macrovascular complications such as coronary heart disease (CHD) and stroke [30]. Epidemiological studies indicate a progressive increase in CHD risk as the serum TC exceeds 5.0 mmol/L [31]. It is thus generally recognized and recommended that treatment of hypertension should, in addition to lowering blood pressure, target correction of dyslipidemia (as well as other CVD risk factors) if present, to reduce overall CVD risk and increase the cost-effectiveness of therapy.

Isolated low HDL-C was the most common individual lipid abnormality among the study participants especially in the controls among whom it represented 71.4% of all forms of dyslipidaemia. Akintunde [26] had earlier reported a similar finding in Osogbo. Odenigbo et al. [33] reported a high rate of low HDL-C among apparently healthy professionals in Asaba, a town which is located in close proximity to Nnewi, our study location. The ATP III guidelines recognize isolated HDL-C as a distinct form of atherogenic dyslipidaemia but state that it is not common in the general population. Our data and those of Odenigbo et al. [33] however suggest that isolated low HDL-C may be a relatively common baseline lipid abnormality among the general population in this part of Nigeria and that the presence of hypertension only escalates it. HDL-C can result in endothelial damage and trigger an increase in BP. The exact mechanism by which a low HDL-C increases CVD risk has however not been fully elucidated, though experimental studies suggest a direct role for HDL-C in promoting cholesterol efflux (this is called reverse cholesterol transport) from foam cells in the atherosclerotic plaque depots in blood vessels to the liver for excretion. HDL-C also exhibits potent anti-inflammatory and antioxidant effects that inhibit the atherogenic process [34, 35]. It has additionally been shown that a low HDL-C level correlates with the presence of other atherogenic risk factor (some of which are emerging risk factors not considered separately during prevalence). According to Pavithran et al. [25] alteration in lipid metabolism including a decrease in HDL-C can result in endothelial damage and trigger an increase in blood pressure which may partially account for its strong predictive power for CHD.

It has long been known that a low level of HDL cholesterol is a powerful predictor of increased cardiovascular risk [36–39]. Eapen et al. [40] showed that male and female patients with low HDL-C levels(<35 mg/dL) and with normal total cholesterol levels have more cardiovascular events (such as heart attacks and unstable chest pain) as compared to their adult counterparts with high HDL-C levels. There is strong epidemiological evidence that low HDL-C is an independent risk factor for CVD [36, 38] with strong suggestions that interventions to increase HDL-cholesterol will yield clinically significant outcome benefits. The Multiple Risk Factor Intervention Trial [41] showed that each decrease in HDL-cholesterol of 1 mg/dL (0.03 mmol/L) was associated with an increase in the risk of coronary heart disease of 2% in men and 3% in women. It has been shown that a 1% reduction in HDL-C is associated with a 2-3% increase in CHD risk. Mounting clinical and experimental evidence show that HDL-Cs exert multiple antiatherogenic and antithrombotic effects that together are consistent with a marked reduction in the risk of a morbid cardiovascular event, supporting an anti-atherogenic role for HDL-cholesterol [41, 42]. In recognition of its status as a CVD risk factor, ATP III recommends that a low HDL-C (≤40 mg/dL which is equivalent to ≤1.04 mmol/L for both men and women) should be a secondary target of therapy aimed at lipid lowering to reduce CVD risk. However several studies have not borne this out [43, 44]. 

 Hypertension and dyslipidaemia are well known to frequently coexist. The coexistence of hypertension and dyslipidaemia has multidimensional clinical implications. First, CVD risk is synergistically enhanced and for this reason, both conditions should be treated aggressively [2]. This association has been linked to background central obesity and consequent insulin resistance which are underlying factors that play major roles in the pathogenesis of both hypertension and dyslipidaemia. The results of a 7 year follow-up study on Finnish men suggested that dyslipidemia characteristic of the metabolic syndrome predicted the development of hypertension [45]. Halperin et al. [3] had also shown that dyslipidemia in apparently healthy individuals leads to hypertension. Hausmann et al. [46] in their intravascular ultrasound studies demonstrated that patients with low HDL cholesterol and high TG levels have more extensive coronary atheromas than those with an isolated elevation of LDL cholesterol. 

Finally, despite the relatively low incidence and burden of coronary heart disease risk factors in black Africans, high-risk groups such as hypertensives may need to be more fully evaluated for lipid abnormalities and therapy initiated early for those found with lipid abnormalities.

## 6. Limitations of the Study

Our study has several limitations. One study limitation is the fact that our study did not collect data from all parts of the country and at best it could only be speculated whether observed relationship is similar all over the country. Secondly being a cross-sectional study by design it cannot observe prospectively and thus cannot associate causal relationships between the factors under study. Finally it is a hospital based study and may not truly represent the population at large as the risk profile of those who did not come to hospital may differ from those who did.

## 7. Conclusion

This study has shown that lipid abnormalities are highly prevalent among newly diagnosed hypertensive patients in South East Nigeria. Efforts should therefore be intensified to fully evaluate Nigerians with hypertension from a lipid and lipoprotein standpoint, and any abnormalities detected are to be taken into consideration during therapy of this group of high-risk patients.

## Figures and Tables

**Table 1:** Shows the demographic, clinical, and biochemical profile of the subjects and controls.

Variable	Subjects	Controls	*t*	*P* value
	Mean + SD	Mean + SD		
Age (years)	58.5 ± 12.4	57.8 ± 12.5	−0.6	0.6
SBP (mm Hg)	163.3 ± 18.9	115.5 ± 10.1	−38.9	<0.001
DBP (mm Hg)	99.1 ± 11.7	70.9 ± 9.1	−30	<0.001
BMI (kg/m^2^)	28.8 ± 5.8	26.7 ± 4.4	−4.5	<0.001
WC (cm)	96.4 ± 13.4	90.8 ± 10.8	−5.1	<0.001
WHR	0.97 ± 0.07	0.94 ± 0.06	−4.9	<0.001
FBS (mmol/L)	5.0 ± 1.2	4.6 ± 0.8	−5.3	<0.001
TG	1.23 ± 0.32	1.10 ± 0.24	−5.4	<0.001
HDL-C	1.25 ± 0.27	1.24 ± 0.57	−0.3	=0.8
LDL-C	3.00 ± 0.82	2.44 ± 0.53	−9.1	<0.001
TC	4.83 ± 0.95	4.15 ± 0.57	−9.7	<0.001

SBP: systolic blood pressure; DBP: diastolic blood pressure; BMI: body mass index; WC: waist hip ratio; FGS: fasting blood sugar; TG: triglycerides; HDL-C: high density lipoprotein cholesterol; LDL-C: low density lipoprotein cholesterol; TC: total cholesterol.

**Table 2:** Prevalence of various serum lipid abnormalities among the study participants.

Lipid abnormality	hypertensives	controls
	*n* (%)	*n* (%)
Elevated TC (≥5.2 mmol/L)	89 (35.6)	16 (6.4)
Elevated LDL-C (≥3.4 mmol/L)	71 (28.4)	15 (6.0)
Elevated TG (≥1.7 mmol/L)	16 (6.4)	2 (0.8)
Low HDL-C (<1.04 mmol/L)	54 (21.6)	40 (16.0)
No lipid abnormality	119 (47.6)	194 (77.6)
One lipid abnormality	54 (21.6)	41 (16.4)
≥2 lipid abnormality	77 (30.8)	15 (6.0)
At least one abnormality	131 (52.4)	56 (22.4)

TG: triglycerides; HDL-C: high density lipoprotein cholesterol; LDL-C: low density lipoprotein cholesterol; TC: total cholesterol.

**Table 3:** Showing Spearman's correlations between lipid profile and clinical parameters.

	TC		LDL-C		HDL-C		TG	
	Subjects	Controls	Subjects	Controls	Subjects	Controls	Subjects	Controls
SBP	−0.032	−0.047	−0.013	−0.041	−0.034	0.101	0.003	−0.007
DBP	−0.054	−0.041	−0.042	−0.007	−.066	0.055	−0.015	−0.036
BMI	0.176**	0.061	0.176**	0.067	−0.001	0.004	0.288**	0.026
WC	0.102	0.081	0.097	0.075	−0.241**	−0.041	0.293**	0.042
WHR	−0.101	0.013	−0.087	0.052	−0.063	−0.117	0.120	−0.038
FBS	0.168**	0.54	0.172**	0.009	−0.115	0.029	0.136*	0.06

*Correlation significant at *P* = 0.05; **Correlation significant at *P* = 0.001; TG: triglycerides; HDL-C: high density lipoprotein cholesterol; LDL-C: low density lipoprotein cholesterol; TC: total cholesterol.

**Table 4:** Shows the multiple regression analysis with total cholesterol, HDL-C, LDL-C, and triglycerides as dependent variables and BP, age, BMI, sex, and FBG as independent variables.

	Unstandardized coeff		Standardized coeff	*t*	sig
	*B*	Std error			
Triglycerides: F5,494 = 7.741 Adjusted *R* = .063*P* < 0.0001					

Constant	.518	.112		4.630	.000
SBP	.001	.000	.148	3.257	.001**
BMI	.008	.002	.150	3.337	.001**
Age	.002	.001	.088	2.012	.045*
FBG	.020	.013	.070	1.572	.117
Sex	.009	.025	.015	.342	.732

LDL-C: F5,494 = 23.245 Adjusted *R* = .182 *P* < 0.0001					

Constant	.702	.270		2.602	.010
SBP	.006	.001	−.248	5.830	.000**
BMI	.011	.006	.080	1.908	.057
Age	.006	.002	.095	2.338	.020*
FBG	.137	.031	.185	4.432	.000**
Sex	−.285	.061	−.191	4.644	.000**

HDL-C: F5,494 = 2.286 Adjusted *R* = .013 *P* < 0.0001					

Constant	1.339	.176		7.628	.000
SBP	.000	.001	.030	.642	.521
BMI	−.004	.004	−.047	−1.025	.306
Age	.000	.002	.005	.103	.918
FBG	.003	.020	.007	.150	.881
Sex	.131	.040	−.148	−3.285	.001**

Total Cholesterol: F5,494 = 26.171 Adjusted *R* = .201					

Constant	2.162	.305		7.081	.000
SBP	.008	.001	.267	6.362	.000**
BMI	.013	.007	.077	1.856	.064
Age	.007	.003	.101	2.514	.012*
FBG	.147	.035	.174	4.211	.000**
Sex	−.373	.069	.218	−5.373	.000**

LDL-C: low density lipoprotein cholesterol; HDL-C: high density lipoprotein cholesterol; SBP: Systolic blood pressure; BMI: body mass index; FBG: fasting blood glucose; coeff: coefficient; Std: standard. *P < 0.05. **P < 0.001.
